# The Future of Biohybrid Regenerative Bioelectronics

**DOI:** 10.1002/adma.202408308

**Published:** 2024-11-20

**Authors:** Alejandro Carnicer‐Lombarte, George G. Malliaras, Damiano G. Barone

**Affiliations:** ^1^ Department of Engineering Electrical Engineering Division University of Cambridge Cambridge CB3 0FA UK; ^2^ Department of Neurosurgery, Houston Methodist Houston 77030 USA; ^3^ Department of Clinical Neurosciences University of Cambridge Cambridge CB2 0QQ UK

**Keywords:** biohybrid interfaces, cell transplantation, implantable devices, neural interfaces, regenerative bioelectronics

## Abstract

Biohybrid regenerative bioelectronics are an emerging technology combining implantable devices with cell transplantation. Once implanted, biohybrid regenerative devices integrate with host tissue. The combination of transplant and device provides an avenue to both replace damaged or dysfunctional tissue, and monitor or control its function with high precision. While early challenges in the fusion of the biological and technological components limited development of biohybrid regenerative technologies, progress in the field has resulted in a rapidly increasing number of applications. In this perspective the great potential of this emerging technology for the delivery of therapy is discussed, including both recent research progress and potential new directions. Then the technology barriers are discussed that will need to be addressed to unlock the full potential of biohybrid regenerative devices.

## Graphical abstract

Regenerative biohybrid bioelectronics represent an emerging class of technology combining the therapeutic potential of implantable bioelectronic devices and cell transplantations. In this perspective the future of this class of technology is evaluated, outlining therapeutic targets and applications. It is finished by discussing the technology challenges that remain to be overcome to enable their clinical potential.

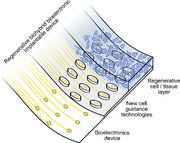

## 1 Introduction

The term “biohybrid” is often used to describe a wide class of technologies that incorporate some biological component, such as cells or tissue‐engineered components. The fields in which biohybrid technologies can be found are extremely varied, ranging from living cell‐actuated robots,^[^ ^1^ ^]^ to microrobot‐modified bacteria^[^ ^2^ ^]^ or synthetic exosomes for drug delivery.^[^ ^3^ ^]^ In the context of implantable bioelectronics, the aim of the “living” components is facilitate the integration of the implant with the host tissue. Whether this integration occurs by growth from the implant, the host, or a combination of both, these characteristics make biohybrid implants particularly well‐suited for regeneration into both healthy and injured tissues. Conceptually, biohybrid implantable technologies have existed for decades. Early forms have included hippocampal neurons housed within silicon probes,^[^ ^4^ ^]^ nerve implants hosting cell populations,^[^ ^5^ ^]^ and nerves transplanted into cortex and electrically interfaced via gold wires.^[^ ^6^ ^]^ While these early implants laid down the foundations for biohybrid technologies – identifying major challenges such as transplant survival, transplant integration with implant, and ethical considerations of this technology – only recently have studies been able to achieve successful the mutual integration between transplanted cellular components and host tissue.

Previously, we have reviewed the past and present of biohybrid bioelectronics.^[^ ^7^ ^]^ In this prior review we described and classified the approaches used to integrate cell and tissue transplantation concepts with implantable bioelectronics to achieve novel ways of interfacing with tissue. This included work from a variety of laboratories, including systems integrating long‐range growing neurons for brain use,^[^ ^8^, ^9^ ^]^ bioresorbable hydrogels encapsulating cells,^[^ ^10^, ^11^ ^]^ and muscle cell layers to host regenerating peripheral nerves.^[^ ^12^, ^13^ ^]^ While much of the work we described in this previous review was conceptual or early‐stage, in the few years from its publication the research in the field has rapidly advanced, and a future in which biohybrid regenerative devices are implemented to treat human disease and dysfunction seems closer than ever. In this perspective, we evaluate the future of biohybrid regenerative bioelectronics, including its potential therapeutic targets and applications and the technological challenges it faces.

## 2 Targets for Biohybrid Technologies as Defined by Clinical Needs

The ability of biohybrid regenerative bioelectronics to integrate with both tissue and electronics at the biological level offers the highest therapeutic potential in applications where technology requires the most intimate interfacing with tissue. While the near infinite number of combinations of different biological transplants and implantable devices offers a great number of potential new biohybrid regenerative technologies, those which fulfill currently unmet clinical needs should have the greatest impact potential and therefore their development should be prioritized.

Implantable technologies are designed to interact with the host tissue by acquiring information from it, modulating its activity, or a combination of both. Irrespective of the modality of this interaction (electrical, chemical, optical, mechanical), a breakdown of the interface between implant and tissue beyond the limit over which communication can occur will result in failure of the technology to meet its therapeutic target. Some degree of interface degradation appears to be inevitable in all implantable bioelectronic applications due to the inflammatory and fibrotic response developing around all implants (known as the foreign body reaction).^[^ ^14^, ^15^, ^16^ ^]^ However, foreign body reaction will affect different devices to a different degree, with technologies designed to interact with ever smaller spatial domains (individual cells or small groups of cells) at the highest temporal resolutions (seconds or milliseconds) suffering the most from degradation in interface performance.

Because of these characteristics the nervous system is a primary potential target for biohybrid bioelectronics. Over the past decades numerous technologies interfacing with the nervous system have emerged, ranging from the restoration of movement and speech in paralyzed patients^[^ ^17^, ^18^ ^]^ to the restoration of sensation in patients with amputated limbs,^[^ ^19^, ^20^ ^]^ with many other implant‐driven treatments currently being explored in preclinical models. This wide range of treatments highlight a common theme: an ever increasing need to interface with the nervous system at the single neuron/axon level, detecting or activating very specific neuronal populations, and with a strong emphasis on temporal salience driven by the millisecond duration of neuronal action potentials and the importance of spike timing in the nervous system. Unfortunately, the decrease in performance following implantation of many high‐resolution technologies driving these treatments is a major barrier to their wider application.^[^ ^15^, ^16^, ^21^ ^]^

The application of biohybrid regenerative bioelectronics to the nervous system offers the potential to address this limitation. While the nervous system has been the focus of most early work in biohybrid regenerative bioelectronics research,^[^ ^7^ ^]^ notable advances have been achieved in recent years. “Living electrodes”, comprising of cultured neurons regenerating through a hydrogel column to integrate with brain tissue^[^ ^8^ ^]^ are a good example of this. The peripheral nervous system in particular has also seen significant progress in biohybrid technology development. A modified version of the “living electrode” device has also been successfully applied in vivo in rodents to bridge and repair injuries in peripheral nerves.^[^ ^22^ ^]^ In our own work, we recently showcased a flexible, muscle cell‐laden device capable of interfacing with peripheral nerves by receiving regenerating axons following an injury in rats. By transplanting a single layer of muscle cells, this device achieved natural amplification of nerve signals, while preserving the high‐resolution recording potential of a sub‐millimeter multielectrode array.^[^ ^23^ ^]^ Beyond testing in preclinical in vivo models, regenerative peripheral nerve interfaces (RPNIs) consisting of isolated muscle tissue monitored by implanted electrodes^[^ ^13^ ^]^ have now been used to control hand prostheses successfully in human patients.^[^ ^24^ ^]^

Skeletal muscle is another attractive target for biohybrid regenerative bioelectronics. Muscle activation is commonly used as a driver of active prostheses in limb amputees.^[^ ^24^, ^25^ ^]^ In electrode‐driven prostheses this typically takes the form of cutaneous electrodes recording muscle activation in the form of electromyogram, which is in turn translated into movement of a prosthesis. Muscle activation, however, occurs in a much more refined manner than surface electrodes can capture, and implanted electrodes able to interrogate individual muscle fibers (beyond the resolution level of the individual motor axon activation) has the potential to greatly increase the active control of prostheses that patients may be able to exert. Combined with its good regenerative capacity, these features make skeletal muscle an attractive target for the development of therapeutically relevant biohybrid regenerative bioelectronic systems. While several aforementioned biohybrid systems designed for peripheral nerve interfacing make use of myocyte cultures to form artificial muscle targets,^[^ ^12^, ^23^ ^]^ efforts have also been made to integrate bioelectronics with host muscle tissue, particularly in the context of targeted muscle reinnervation as a tool to guide prosthetics.^[^ ^26^, ^27^ ^]^ Our lab, for example, has developed collagen‐based bioresorbable gel bioelectronics that integrate with skeletal muscle upon implantation, leading to minimal fibrotic scarring and good signal‐to‐noise ratio recordings.^[^ ^28^ ^]^

The potential for biohybrid regenerative interfaces in other fields is also significant. Gut function, particularly in relation to enteric nervous system (ENS) activity, has gathered significant attention in recent years. However, tools to study ENS function in vivo remain very limited. Regenerative devices capable of integrating into these highly dynamic tissues represent a promising avenue for their study, particularly when both cell transplantation^[^ ^29^ ^]^ and tissue engineering^[^ ^30^ ^]^ have been extensively applied to it. Traditional implant technology has been a poor match for the motility of the gut, but recent work has shown that the use of flexible or stretchable devices can be leveraged to achieve stable interfacing.^[^ ^31^, ^32^, ^33^ ^]^

While technology‐tissue integration is in our view a key benefit of biohybrid regenerative technologies over conventional devices, these systems also offer a second major advantage. Tissue and cell regeneration does not occur at random. Regeneration is driven and guided by a myriad of chemical, mechanical, and electrical cues – which can in turn be manipulated. Biohybrid systems offer the opportunity to tailor the host cell type or components onto which implants connect by enhancing their cell‐specific regeneration. Existing work has only scratched the surface of this potential: transplanted muscle cells selectively receiving motor axons,^[^ ^23^ ^]^ and transplanted motor neurons selectively innervating muscle.^[^ ^22^ ^]^ However, as the technology matures and more avenues for regeneration steering are employed, we expect to see this characteristic of biohybrid regenerative bioelectronic devices become central to their therapeutic potential.

## 3 Advances in Cell Transplantation and Tissue Engineering Can Drive Progress in Biohybrid Regenerative Bioelectronics

Biohybrid regenerative bioelectronics rely on the integration of a transplanted cell population with a host cell population. Therefore, it is unsurprising that advances in our ability to produce better transplant cell technologies and tissue‐engineered constructs can give rise to exciting opportunities in the field of biohybrid bioelectronics. A number of advances in areas such as organoid transplantation and lipid bilayer isolation represent relevant tools that could benefit future biohybrid implant technologies.

Biohybrid regenerative technologies have so far focused on the incorporation of relatively simple biological components into implants. These simpler biological components have ranged from extracellular matrix combinations^[^ ^28^ ^]^ to xenotransplants^[^ ^34^ ^]^ or allotransplants of cells.^[^ ^35^ ^]^ While some work has succeeded in utilizing translationally‐relevant cell populations such as autograft muscle cells^[^ ^12^, ^24^ ^]^ or induced pluripotent stem cells,^[^ ^23^ ^]^ overall the field has focused predominantly on achieving some degree of integration across the three domains of technology, transplant, and host. However, as more biohybrid regenerative bioelectronic technologies are developed and research laboratories overcome methodological limitations expected of a field still in its infancy, more advanced cell and tissue transplants can be expected to become the focus of biohybrid technologies.

The last few years have seen exciting advancements in cell and tissue transplantation. Organoids, in vitro grown cell structures resembling organs in structure and function, have now been transplanted into animals and integrated into tissue including brain,^[^ ^36^, ^37^, ^38^ ^]^ liver,^[^ ^39^, ^40^ ^]^ pancreas,^[^ ^41^ ^]^ and bowel.^[^ ^42^ ^]^ Within the field of cell transplantation, organoids provide an exciting therapeutic opportunity by representing a better replacement for complex structures such as neuronal circuits that may be damaged in injury and disease. These complex structures would otherwise be difficult to reconstruct using simpler transplants such as single cell type suspensions. For biohybrid bioelectronics this offers enormous potential to interface with the body in new ways. In vitro, organoids have been interfaced with technology, enabling monitoring and modulation of circuits at high resolutions using both traditional stiff multielectrode arrays^[^ ^43^, ^44^ ^]^ and newer flexible electrode devices.^[^ ^45^, ^46^ ^]^ It may therefore be expected that in the future we may see biohybrid regenerative interfaces restoring function in the body by, for example, replacing and controlling neural circuitry through an organoid component. This is particularly promising given the interest of the field in the development of organoids combining multiple interconnected tissues, such as brain‐to‐spinal cord connections.^[^ ^47^ ^]^

More generally, there is a great range of biological components that are readily interfaced with in vitro which may offer opportunities for important advances in biohybrid applications. Organs such as gut,^[^ ^48^ ^]^ muscle,^[^ ^49^ ^]^ and blood‐brain barrier^[^ ^50^ ^]^ among many others can be reconstructed through combinations of various cell types and tissue engineering principles and monitored via bioelectronics as done in organ‐on‐a‐chip technologies.^[^ ^51^ ^]^ Induced pluripotent stem cell can be differentiated over short periods of time to produce human transplantation‐ready populations.^[^ ^52^ ^]^ Cell transplantation technologies are also steadily advancing to produce better strategies to enhance transplant survival and integration.^[^ ^53^, ^54^ ^]^ Decades of stem cell therapy research have optimized the harvesting, isolation, and expansion of highly specific cell populations with regenerative therapy potential. The concepts and methodologies of cell and tissue transplantation that biohybrid regenerative bioelectronic technologies can tap into are extremely diverse, highlighting the enormous potential of this emerging field.

Another field that may lead to significant advances in biohybrid regenerative technologies is the isolation and incorporation of subcellular structures to technology. Lipid bilayers, for example, can be constructed or reconstituted onto artificial systems capable of interacting with them.^[^ ^55^, ^56^ ^]^ This has classically been employed as a technique to study the properties of these membranes or perform drug screening. However, as these membranes can contain a wide range of biologically active transmembrane proteins, the potential exists for them to be utilized as a highly customizable layer interfacing tissue with implant in a biohybrid regenerative device. Options among these may be regeneration of axons onto an implant driving the formation of specific types of synapse, an exciting possibility given recent progress in the formation of synaptic structures in Supported Lipid Bilayers (SLBs),^[^ ^57^ ^]^ or the use of lipid bilayers to improve device biocompatibility and tissue integration. On the other hand, SLBs are known to be unstable in less controlled environments, and their use in vivo will likely need to be combined with supportive structures, such as hydrogels.

## 4 Future Technology Development Needed

Despite the potential of biohybrid regenerative bioelectronics as an avenue for therapy, much work remains to be done. While it is becoming increasingly clear that implantable devices containing both electrodes and cell components are now achievable, clinically‐relevant therapies delivered by these will require further advancement of the technology in multiple areas.

The first barrier to biohybrid bioelectronic technology – maintaining a stable and healthy cell transplant‐device connection after implantation – is now successfully being overcome through different approaches. Laboratories such as our own have made use of ultraflexible substrates to ensure cells remain in direct contact with the implanted device despite the mechanically challenging environment of a living, moving body.^[^ ^23^ ^]^ Other groups have made use of highly growth‐prone cells to extend the cell layer into host tissue while leaving the implanted electronics protected,^[^ ^58^ ^]^ or to enable regenerating host cells to approach the implanted device.^[^ ^12^ ^]^

**Figure 1:**
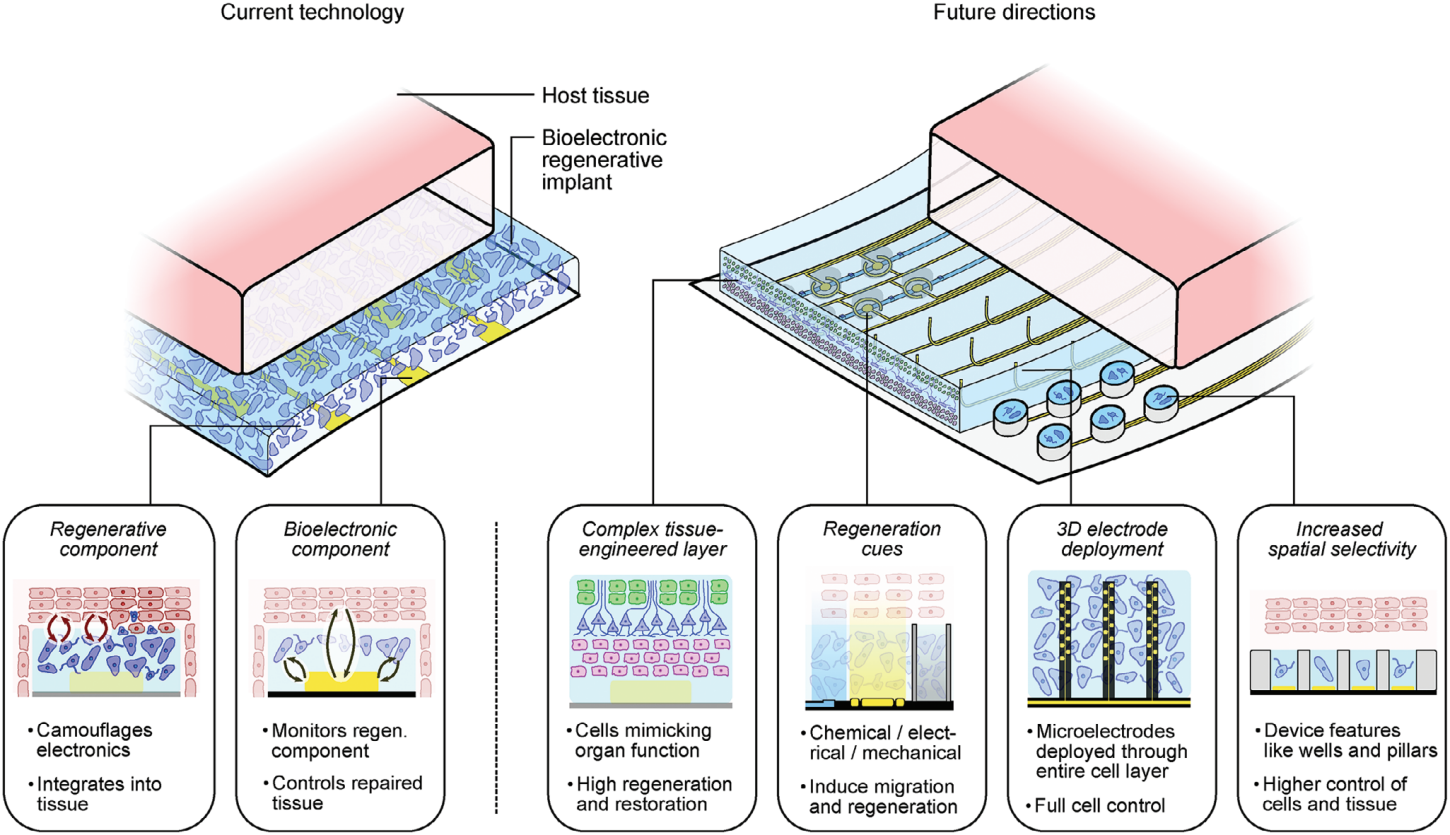
The future of biohybrid regenerative bioelectronics. Features of technology in its current state (left) and proposed future directions (right) improving upon the advantages offered by biohybrid regenerative technologies to unlock new therapeutic opportunities.

In order to realize the potential of regenerative biohybrid implants, however, the technology needs to be further improved to allow for a much better control and guidance of transplanted cells. We can identify four key challenges to drive future biohybrid regenerative technology research (**Figure** **1**). Microelectrode arrays implanted into the brain (a more traditional, non‐biohybrid technology), for example, can record and modulate neuron activity up to the individual neuron level. Currently, biohybrid bioelectronic technologies cannot replicate this level of resolution. The first challenge is the need to improve the spatial selectivity with which devices interface with the transplanted cell population to bring biohybrids to the resolution level of other existing technologies. This translates to being able to pattern engineered tissues commensurate with the size of microelectrode technologies to achieve optimal spatial resolution. Fortunately, there is an abundance of work in the field of organ‐on‐a‐chip and similar research to draw inspiration from. This includes technologies such as the patterning of 3D structures such as microwells and pillars,^[^ ^59^, ^60^ ^]^ spatially‐restricted surface coatings,^[^ ^61^, ^62^, ^63^ ^]^ and compartmentalized or fluidic‐containing systems^[^ ^64^, ^65^, ^66^ ^]^ in order to contain and distribute small cell populations over devices.

Secondly, we need to improve our ability to customize the structure of engineered tissues on biohybrid devices. Most tissues in the body function through a delicately arranged internal structure. Whether constructing engineered neuronal cortex stratified into cortical layers, or gut tissue consisting of muscle, neuronal, and epithelial layers; future regenerative implants will likely need to contain complex tissues mimicking many of the structures they will be replacing in their host. While In vitro work has demonstrated that neural circuits on can be constructed on electrode arrays,^[^ ^67^, ^68^ ^]^ and advances in organoids and their transplantation can be leveraged in this area, engineered tissues will have to be integrated with implantable devices. This will require the adaption of techniques from other fields to accommodate the challenges of implant fabrication and its in vivo environment. Similarly, while organoids or other in vitro technologies typically aim to replicate a mature tissue in a dish, transplanted regenerative biohybrid devices need to integrate with their host tissue. Whether their tissue‐engineered components should be made up of differentiated, non‐differentiated, or a combination of cells at the time of transplantation is another key aspect of future research. Tools such as bioprinting^[^ ^69^, ^70^ ^]^ may offer an effective tool to achieve the required complex tissue design and device integration, also enabling some flexibility of differentiation stage as required. These could in turn benefit from techniques such as spatial multiomics to better characterize and track changes in these customized tissues, as is already being done in fields such as organoid research.^[^ ^71^, ^72^ ^]^ In vivo, devices should also be designed to facilitate tracking of transplant survival and integration. In areas such as the brain, transplants can be tracked by intravital microscopy through a cranial window.^[^ ^38^ ^]^ This could be applied in combination with transparent implant devices^[^ ^73^ ^]^ to allow tracking and characterization of biohybrid regenerative device survival. In other, less accessible areas of the body, the device itself could serve as a platform to track transplant state through acquisition of chemical and electrical signals.

A secondary challenge emerging from the use of increasingly complex engineered tissues will be ensuring their survival once transplanted in vivo. Lacking an immediate blood supply, ischaemia can prevent transplanted tissues from surviving for long enough to integrate with the host tissue. Inflammation‐derived damage to the transplant can also disrupt the fragile architecture of an engineered tissue. Strategies to control these limitations – some derived from the field of tissue engineering such as engineering microvasculature,^[^ ^74^ ^]^ others from reconstructive surgery techniques such as promotion of angiogenesis and dampening of inflammation^[^ ^75^ ^]^ – may represent an important focus of future work.

Thirdly, implant multielectrode arrays will have to be adapted to integrate with the more complex engineered tissues. Key among these adaptations will be the introduction of 3D electrode arrays in biohybrid regenerative devices. Deploying microelectrodes across not only multiple locations, but also multiple layers of the biological component of the implant will likely be necessary to fully monitor the tissue‐engineered construct and the host tissue in certain applications. 3D multielectrode array architectures are already being developed for implantable devices with form factors such as threads and nets.^[^ ^76^, ^77^, ^78^ ^]^ However, these will likely need to be adapted and improved to support the growth of the biohybrid cell or tissue layer in vitro before transplantation. While organ‐on‐a‐chip devices may offer guidance on how to do this, it is important to highlight that good adherence of cells and tissue compatibility following in vivo transplantation requires the use of technologies that trigger low foreign body reaction (e.g., soft or flexible materials mechanically matched to tissue).^[^ ^16^, ^79^ ^]^ This design criterion does not apply to in vitro‐specific technologies, so 3D organ‐on‐a‐chip device designs may not always be readily translated to biohybrid regenerative devices.

Lastly, on the in vivo side, the guidance of regeneration and cell integration will likely be a key development that will greatly benefit biohybrid regenerative technologies. Research so far has achieved implant‐host integration through the intrinsic regenerative capacity of either injured host tissue^[^ ^12^, ^23^, ^28^ ^]^ or transplanted cells.^[^ ^22^, ^58^ ^]^ However, there already exists an enormous body of research on the enhancement and guidance of tissue regeneration. Driving regeneration and integration of devices with tissue has the potential to greatly expand biohybrid technology capabilities by, for example, allowing devices to connect to a larger volume of host tissue, or enabling devices to connect to specific cell types to achieve selectivity in their interfacing. The presence of an implant within the biohybrid construct in itself provides a useful platform for the local delivery of cues to guide regeneration. We therefore expect future biohybrid regenerative bioelectronics research to develop the capabilities for this delivery, particularly in three specific areas: chemical, electrical, and mechanical cues.

Chemical cues have for long been considered primary mediators of cell behavior in regeneration, and have been utilized to enhance tissue regeneration through local delivery – for example in the nervous system.^[^ ^80^, ^81^, ^82^, ^83^ ^]^ Implantable devices can be modified to incorporate microfluidic drug delivery systems through mechanisms such as convection‐enhanced delivery^[^ ^84^, ^85^, ^86^ ^]^ and electrically‐driven drug delivery.^[^ ^87^, ^88^, ^89^, ^90^ ^]^ Molecules such as chondroitinase to digest glial scar‐associated inhibitory factors following injury and facilitate regeneration in the spinal cord,^[^ ^91^, ^92^ ^]^ or neurotrophic growth factors to encourage and guide axonal growth^[^ ^83^, ^93^ ^]^ – all with substantial research supporting their effectiveness in vivo – may be good candidates for delivery in biohybrid implants, particularly in the context of nervous system integration. Beyond well tested regeneration‐associated molecules, newer tools such as RNA,^[^ ^94^ ^]^ extracellular vesicles,^[^ ^95^ ^]^ and nanoparticles^[^ ^96^, ^97^ ^]^ offer unprecedented opportunities to modulate cell before or after transplantation. In the future we may expect microfluidic drug delivery to become an essential component to guide biohybrid implant regeneration in vivo.

Mechanical cues provide a similarly powerful avenue to guide regeneration of cells in biohybrid devices. It is well established that many cell types respond to the mechanical properties of their environment, a process known as mechanotransduction.^[^ ^98^ ^]^ Cell migration and growth, in particular, are strongly guided by factors such as the stiffness of their substrate. In optic tract development, for example, small differences in substrate stiffness have been observed to be sufficient to steer axons to their correct destination in the brain.^[^ ^99^ ^]^ In implantable devices, certain surface properties such as stiffness^[^ ^100^ ^]^ and roughness^[^ ^101^ ^]^ have been shown to modulate host interaction with the implant at the cellular level. Features such as ridges and grooves, as well as other topographical cues are also well known to guide cellular processes such as axon growth.^[^ ^102^, ^103^, ^104^, ^105^ ^]^ While mechanical barriers (e.g., agarose column walls) have been implemented in existing biohybrid regenerative systems to guide regeneration into tissue,^[^ ^58^ ^]^ future devices may be further modified mechanically to provide sites or structural features promoting host or transplant cell attachment, growth, or avoidance.

Electrical stimulation and electric fields are also known to strongly influence cell behavior, including regeneration. While this domain has been substantially less explored than chemical delivery in research, likely in part due to the difficulty to deliver electrical cues locally, once again the presence of an implant containing microelectrodes provides a great platform for their implementation in biohybrid bioelectronic implants. AC stimulation of tissue is known to have a range of biological effects. In both animal models and humans, brief AC stimulation has been used to enhance nerve regeneration and functional recovery^[^ ^106^, ^107^ ^]^ – an effect thought to arise from increased expression of the neurotrophin BDNF and its receptor driving a pro‐regenerative axon phenotype.^[^ ^108^ ^]^ DC stimulation, while more difficult to deliver using conventional electrode technologies, strongly influences cell migration, axon sprouting, and guided growth, and has been utilized to for example drive axon regeneration in spinal cord injury.^[^ ^109^, ^110^ ^]^ The implementation of electrical stimulation to influence cell behavior over long time scales is a growing area of research, particularly for application in stem cell differentiation^[^ ^111^ ^]^ and engineering of tissues such as bone^[^ ^112^ ^]^ or cardiomyocytes.^[^ ^113^ ^]^ While significant work remains to be done, such as identification of new biological effects and effective stimulation paradigms, better spatial localization of stimulation, and improvement of electrode materials for stable long term stimulation delivery; it appears clear that electrical stimulation is a powerful tool that may see use in driving regeneration in the future biohybrid bioelectronics.

Although we have here mostly focused on the technology advances needed to drive the development of biohybrid regenerative bioelectronics, their translation into clinical use will also require addressing important regulatory and industry challenges. Implantable medical devices^[^ ^114^, ^115^ ^]^ and cell transplantation^[^ ^116^, ^117^ ^]^ therapies are regulated by separate processes in many countries, which lay out the path these must follow to reach the market for widespread use in humans. The combined use of these two implant and transplant modalities in the form of regenerative biohybrid bioelectronics poses a significant problem at the regulatory level as generally neither route may be well‐equipped to handle bringing these to market. Commercialization of these technologies will also require the development of fabrication pipelines capable of supporting these combined modalities. This can bring important new challenges, such as the need to store and provide implantable devices under sterile conditions while also preventing them from drying out and damaging the cellular portion of the device. While the exact nature of the challenges and solutions will likely vary by country and type of implant, it seems likely that the future of regenerative biohybrid bioelectronics will require the involvement of policymakers and industry stakeholders to ensure their successful translation into clinical use.

## 5 Conclusion

Biohybrid regenerative bioelectronics is a growing area of research with great therapeutic potential. Efforts from multiple labs over the last few years have demonstrated that the combination of implanted bioelectronics, cell transplantation, and tissue regeneration can now be achieved. While the technology is still at its infancy and remains far from reaching human clinical use, biohybrid regenerative bioelectronics can leverage the abundant research in both the fields of cell transplantation and implant technology. The wide therapeutic potential of biohybrid regenerative bioelectronics makes it a technology well worth developing, and a uniquely exciting opportunity to improve the lives of patients.

## Conflict of Interest

The authors declare no conflict of interests.
